# Genetic Characteristics and Transcriptional Regulation of Sodium Channel Related Genes in Chinese Patients With Brugada Syndrome

**DOI:** 10.3389/fcvm.2021.714844

**Published:** 2021-08-05

**Authors:** Ziguan Zhang, Hongwei Chen, Wenbo Chen, Zhenghao Zhang, Runjing Li, Jiajia Xu, Cui Yang, Minwei Chen, Shixiao Liu, Yanling Li, TzungDau Wang, Xin Tu, Zhengrong Huang

**Affiliations:** ^1^Department of Cardiology, Xiamen Key Laboratory of Cardiac Electrophysiology, Xiamen Institute of Cardiovascular Diseases, The First Affiliated Hospital of Xiamen University, School of Medicine, Xiamen University, Xiamen, China; ^2^Department of Internal Medicine, Cardiovascular Center and Division of Cardiology, National Taiwan University Hospital and National Taiwan University College of Medicine, Taipei, China; ^3^Cardio-X Center, Key Laboratory of Molecular Biophysics of the Ministry of Education, College of Life Science and Technology and Center for Human Genome Research, Huazhong University of Science and Technology, Wuhan, China

**Keywords:** brugada, SCN5A, China, genetic characteristics, post-transcriptional modifications

## Abstract

**Objective:** To investigate the genetic characteristics and transcriptional regulation of the SCN5A gene of Brugada syndrome (BrS) patients in China.

**Methods:** Using PubMed, Medline, China National Knowledge Internet (CNKI), and Wanfang Database, Chinese patients with BrS who underwent SCN5A gene testing were studied.

**Results:** A total of 27 suitable studies involving Chinese BrS patients who underwent the SCN5A gene test were included. A total of 55 SCN5A gene mutations/variations were reported in Chinese BrS patients, including 10 from southern China and 45 from northern China. Mutations/variations of BrS patients from southern China mostly occurred in the regions of the α-subunit of Nav1.5, including DIII (Domain III), DIV, DIII-DIV, C-terminus regions, and the 3'UTR region. Furthermore, we analyzed the post-transcriptional modifications (PTMs) throughout the Nav1.5 protein encoded by SCN5A and found that the PTM changes happened in 72.7% of BrS patients from southern China and 26.7% from northern China.

**Conclusions:** SCN5A mutations/variations of BrS patients in southern China mostly occurred in the DIII-DIV to C-terminus region and the 3'-UTR region of the SCN5A gene, different from northern China. PTM changes were consistent with the mutation/variation distribution of SCN5A, which might be involved in the regulation of the pathogenesis of BrS patients.

## Introduction

Brugada syndrome (BrS) is an inheritable arrhythmogenic disease. The typical electrocardiographic manifestations include ST segment elevation ≥2 mm and T wave inversion on the right thoracic lead (V1–V3) of ECG. BrS is prone to polymorphic ventricular tachycardia, ventricular fibrillation, and sudden cardiac death while the heart structure is normal (1, 2). It is more prevalent in Asian population with an onset age of 30–40 years old, and the ratio of male to female is 8–10:1 (3, 4). The mortality accounted for 4–12% of sudden death each year and even for 20% sudden death without organic heart disease in Southeast Asia (5).

Up to date, 23 genes have been confirmed to be related to BrS (5–7), including gene mutations/variations that lead to ion channel dysfunction such as sodium, calcium, and potassium ions. About 30–35% of BrS patients were identified with pathogenic mutant genes, while SCN5A mutations/variations encoding the Nav1.5 α-subunit of the cardiac sodium channel accounted for 20 to 30% (5, 8–10). Currently, the recommended treatment for BrS is ICD implantation and medication (quinidine, isoproterenol, etc.); however, therapeutic effect is unsatisfactory.

The mutations/variations from BrS patients can significantly reduce the inward Na^+^ current by delaying activation, accelerating inactivation, delaying reactivation, or reducing the membrane expression of ion channels (11, 12). Furthermore, the decrease in the inward Na^+^ current has influence on the depolarization and repolarization of the cardiac action potential, thus causing the generation of typical ECG of BrS (2). Genetic distribution characteristics of BrS on the SCN5A mutations/variations from the world (9) and Japan (13) had been reported. However, there are no genetic distribution analyses on the SCN5A mutation/variation location sites in Chinese BrS patients until now. In the present study, we aim to analyze the reported SCN5A mutation/variation location sites of Chinese BrS patients and predict the PTMs affected by the mutations/variations.

## Materials and Methods

### Information Retrieval and Inclusion Criteria

Two investigators searched Medline, PubMed, CNKI, and Wanfang Database. The query terms were “Brugada syndrome” “China” and “SCN5A.” Articles published in Chinese or English in peer-reviewed journals that met the following criteria were included in our study.

A. Inclusion of subjects with BrS were as previously defined (14).
B. Chinese patients.
C. Who underwent SCN5A gene DNA sequencing.

In addition, we also contacted the corresponding author of several studies in order to obtain more specific experimental data that were not included in the article. In order to resolve any difference or uncertainty between the two investigators, a third investigator was responsible for reexamining the source data and consultation.

### Predictive Analysis the PTMs of SCN5A

First, we retrieved the amino acid sequence and analyzed the domain details of the α-subunit of SCN5A protein in the following website: https://www.uniprot.org/uniprot/Q14524, then predicted the PTM sites with the software on the amino acid sites and mapped on the mutation/variation sites reported from the literature in China. Furthermore, we analyzed the change of PTMs in the natural variant.

### Statistical Analysis

Categorical variables were expressed as percentage and analyzed using the chi-square test. All analyses were conducted using SPSS version 17.0 (SPSS Inc., Chicago, IL, USA). Statistical significance was set at *p* < 0.05.

## Results

### Studies Retrieved and Information Extraction, and Distribution of the SCN5A Mutations/Variations on Nav1.5 Protein

**Figure 1:**
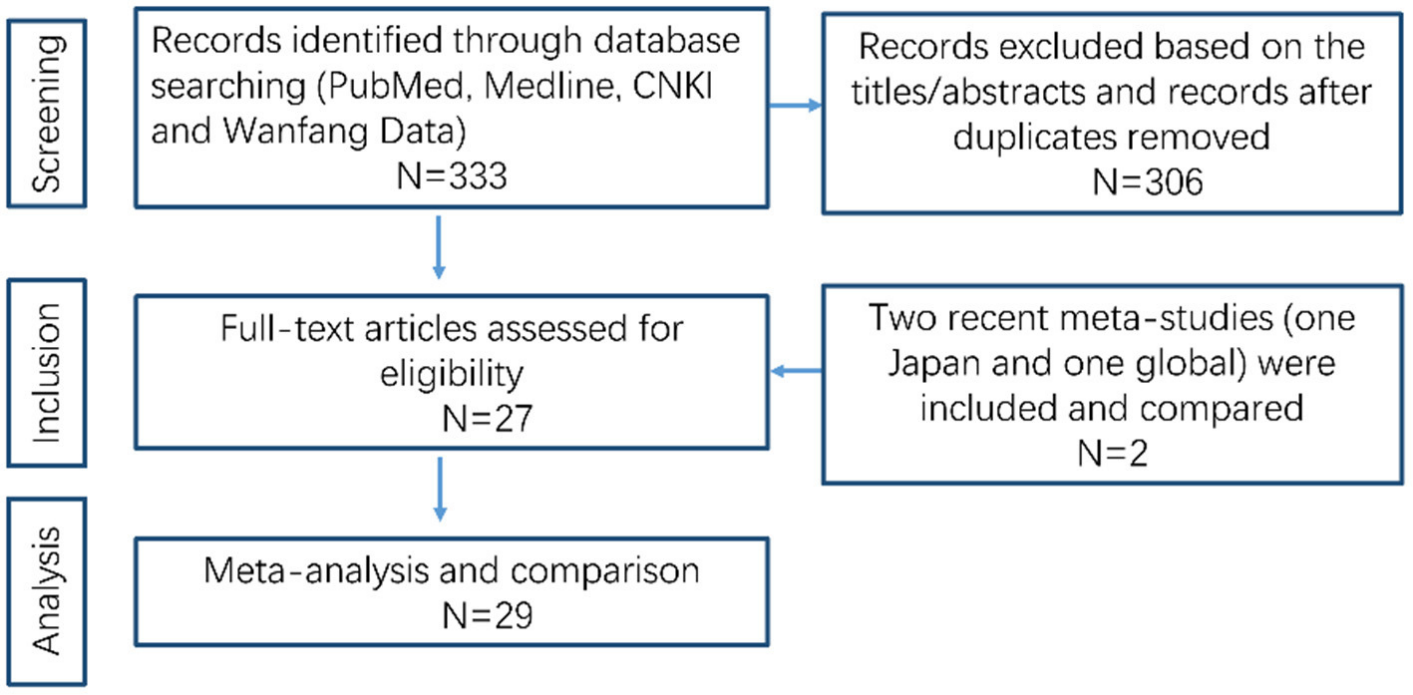
Flow diagram of data search and collection.

A flow chart of the data research is shown in Figure 1. We excluded 306 unqualified studies that did not match the inclusion criteria or were duplicates. A total of 27 suitable studies were included, and details are shown in Table 1. Duplicated loci were removed, and a total of 55 mutations/variations including 45 sites from northern China and 10 sites from southern China (Guangdong, Guangxi, Hong Kong, Hainan, Jiangxi, Fujian, Taiwan) were included. Further analysis of these loci was performed as detailed below.

**Table 1:** Distribution characteristics of SCN5A gene mutation/variation sites in Chinese BrS patients.

**Location**	**Nucleotide change**	**Amino acid change**	**Structural position**	**Investigator**
China (Guangxi)		G1712C	DIV S5-S6	Chen et al. (15) Ruan et al. (16)
China (Guangxi)	A5471G	N1774S	C terminus	Ren et al. (17)
China		A29A	N terminus	Liu et al. (18)
China		E1061E	DII-DIII	Liu et al. (18)
China		D1818D	C terminus	Liu et al. (18)
China		6365a>G 3'UTG		Liu et al. (18)
China		7204t>A 3'UTG		Liu et al. (18)
China		7205c>T 3'UTG		Liu et al. (18)
China	G3578A	R1193Q	DII-DIII	Liu et al. (18)
South China	4087insC		DIIIS4change, DIIIS5-S6 and DIV missing	Chen et al. (19)
China		C1363F	DIII S5-S6	Yan et al. (20)
China	G283A	V95I	N terminus	Liang et al. (21)
China	C4946T	A1649V	DIV S4-S5	Liang et al. (21)
China	Exon 28 missing TCT	del1617	DIV S3-S4	Liang et al. (21)
China (Hebei)	C5457T	A1818A	C terminus	Tian et al. (22)
China		R1913C	C terminus	Qiu et al. (23)
China	G87A		N terminus	Qiu et al. (23)
China	703+130G>A		Intron 6	Qiu et al. (23)
China	1143-3C>A		Intron 9	Qiu et al. (23)
China	A1673G	H588R	DI-DII	Qiu et al. (23); Tian et al. (24); Yi et al. (25)
China	G3578A	R1193Q	DII-DIII	Qiu et al. (23)
China	3840+73G>A		Intron 21	Qiu et al. (23)
China	4245+81G>T		Intron 23	Qiu et al. (23)
China	4245+82A>G	L1291L	Intron 23	Qiu et al. (23)
China	4299+83T>C		Intron 24	Qiu et al. (23)
China	T5457C		C terminus	Qiu et al. (23)
China (Gansu)	G292A	G98R	N terminus	Gong et al. (26)
China (Hebei)	C3549T	T1183T	DII-DIII	Tian et al. (27)
China		K317N	DIS5-S6	Yi et al. (25)
China (Xinjiang or Shanxi)		R230Q	DIS4	Li et al. (28)
China (Xinjiang or Shanxi)		V469V	DI-DII	Li et al. (28)
China (Xinjiang or Shanxi)		R511K	DI-DII	Li et al. (28)
China (Xinjiang or Shanxi)		V522A	DI-DII	Li et al. (28)
China (Xinjiang or Shanxi)		K698N	DI-DII	Li et al. (28)
China (Xinjiang or Shanxi)		G878G	DII S5-S6	Li et al. (28)
China (Xinjiang or Shanxi)	T909	M303T	DIS5-S6	Qin et al. (29)
China (Fujian)	c.4886G>A	R1629Q	DIV S4	Zeng et al. (30)
China (Fujian)	C6995T		3'UTR	Zhao et al. (31)
China (Fujian)	c.5262G>A	D1690N	DIV S5-S6	Zeng et al. (32)
China		Q55X	N terminus	Teng et al. (33)
China		R535X	DI-DII	Teng et al. (33)
China		W822X	DII S4	Teng et al. (33)
China		G867X	DII S5-S6	Teng et al. (33)
China		R1623X	DIV S4	Teng et al. (33)
China		S1812X	C terminus	Teng et al. (33)
China		Q1118X	DII-DIII	Teng et al. (33)
South China	c.1198G>A	p.Giy400Arg	DI S6	Zhang et al. (34)
South China	c.4282G>T	p.Ala1428Ser	DIII S5-S6	Zhang et al. (34)
South China	c.5676delC	p.Thr1893Profs*29	C terminus	Zhang et al. (34)
South China	c.5692C>T	p.Arg1898Cys	C terminus	Zhang et al. (34)
China	c.1960G>T	p.E654X	DI-DII	Liu et al. (35)
China	1651G>A	A551T	DI-DII	Chiang et al. (36)
China (Hubei)	c.4282G>T	p.A1428S	DIII S5-S6	Zhu et al. (37)
China	3578G>A	R1193Q	DIII-DIV	Li et al. (38)
China	3269C>T	P1090l	DII-DIII	Juang et al. (39)
China	1776C>G	N592K	DI-DII	Juang et al. (39)
China (Taiwan)	rs11708996 G>C		Intron	Juang et al. (40)
China		A226V	DIS4	Mok et al. (41)
China		H681P	DI-DII	Mok et al. (41)
China		R1193Q	DII-DIII	Mok et al. (41)
China		V1951L	C terminus	Mok et al. (41)

**Figure 2:**
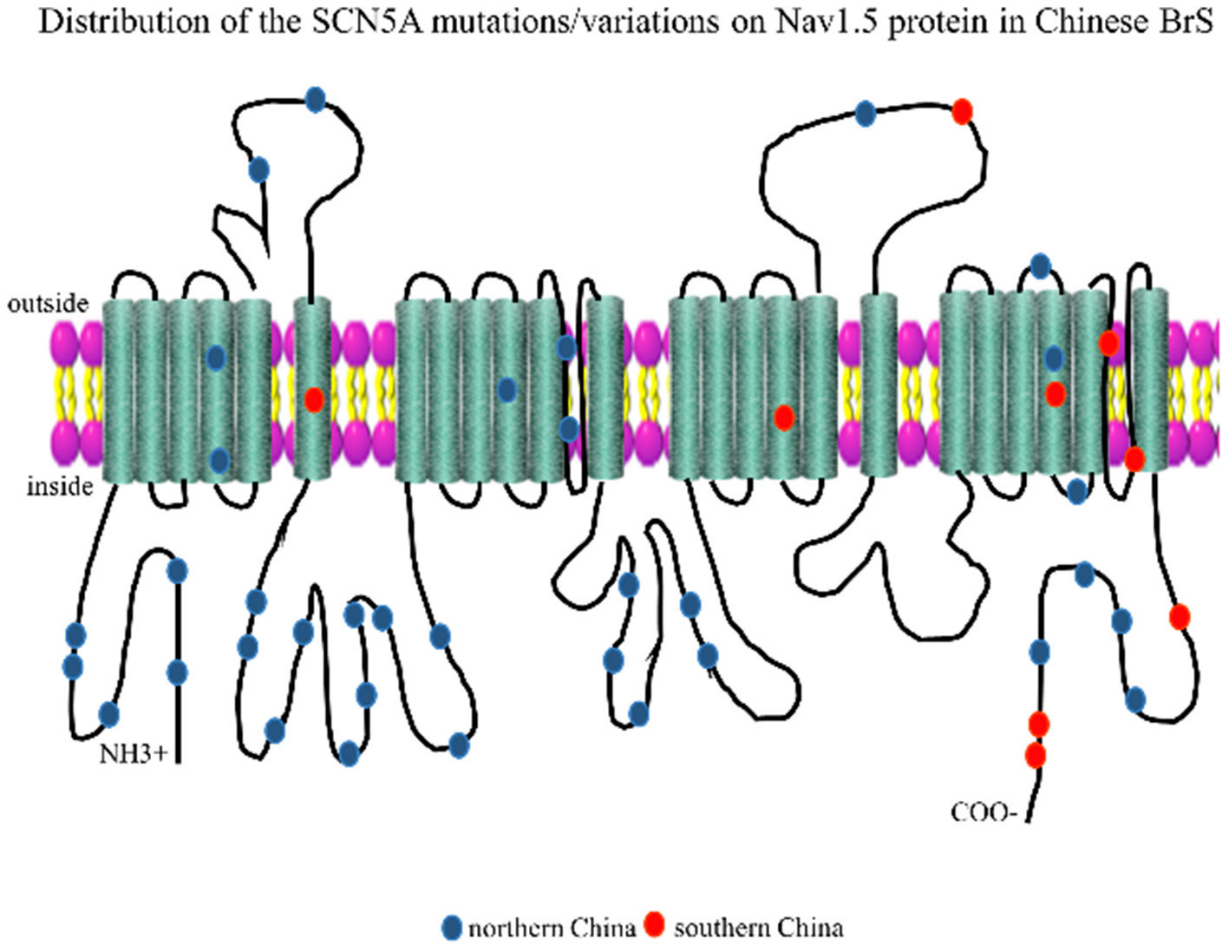
Representation of SCN5A gene mutations/variations was labeled on the corresponding location of the Nav1.5 protein structure. The blue dots represented mutations/variations from southern China. The red dots represented mutations/variations from northern China.

The Nav1.5 channel has four highly conserved homologous transmembrane-spanning domains (DI–DIV) that are connected by an interdomain linker (IDL), and each domain consists of six transmembrane α spins (S1–S6). In order to visualize the distribution of these loci, we marked the mutation/variation sites on Nav1.5 protein (excluding introns and 3'UTR, which were not translated into amino acids), as shown in Figure 2. The blue dots represented the SCN5A mutations/variations from northern China, and the red dots represented the SCN5A mutations/variations from southern China.

### Locational Distribution of SCN5A Gene Mutations/Variations in China

**Figure 3:**
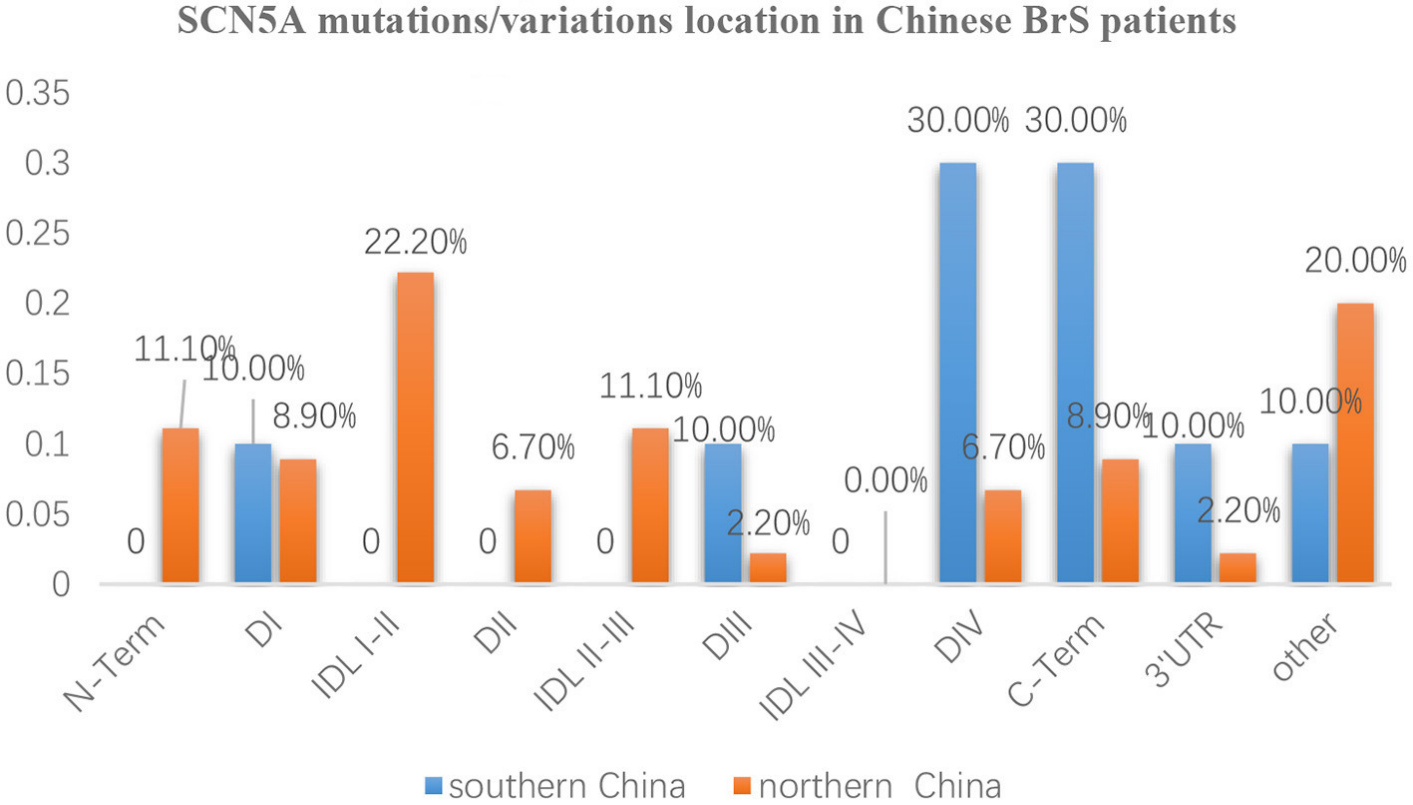
The distribution characteristics of mutation/variation sites in different parts of the SCN5A gene in Chinese BrS patients The blue columns represent the distribution of gene mutations/variations from southern China. The orange columns represent the distribution of gene mutations/variations from northern China.

The whole SCN5A gene was divided into 5′-UTR, N-Term (N-terminus), D I, IDL I-II, DII, IDL II-III, DIII, IDL III-IV, DIV, C-Term (C-terminus), 3′-UTR, and other regions. Mutation/variation loci distribution is shown in Figure 3. Distinguished by those 12 parts, the distribution in southern China and northern China were 5′-UTR (0 vs. 0), N-Term (0 vs. 11.1%), DI (10 vs. 8.9%), IDL I-II (0 vs. 22.2%), DII (0 vs. 6.7%), IDL II-III (0 vs. 11.1%), DIII (10 vs. 2.2%), IDL III-IV (0 vs. 0), DIV (30 vs. 6.7%), C-Term (30 vs. 8.9%), 3′-UTR (10 vs. 2.2%), and other (10 vs. 20%), respectively. It was found that SCN5A mutations/variations in northern China were mainly concentrated (60%) in 5′-UTR, N-Term, DI, IDL I-II, DII, and IDLII-III while mostly distributed (80%) in DIII, IDL III-IV, DIV, C-Term, and 3′-UTR in southern China.

**Figure 4:**
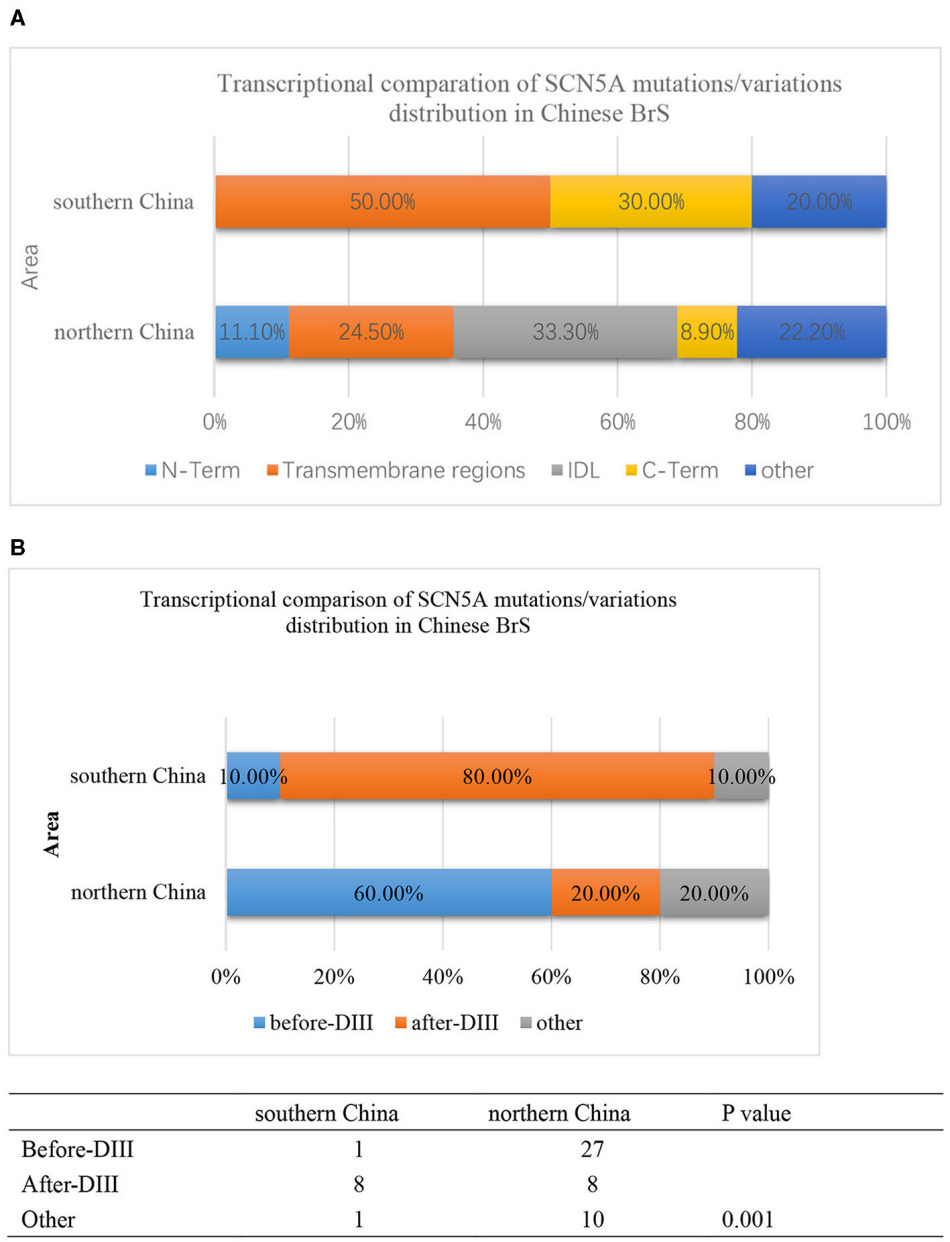
**(A)** The transcriptional comparison of the SCN5A mutation/variation distribution between southern China and northern China, which was distinguished by N-term, Transmembrane, IDL, C-term, and other parts of the SCN5A gene. The light blue bar represents N-terminus. The orange bar represents transmembrane regions. The gray bar represents the IDL region. The yellow bar represents the C-terminus. The deep blue bar represents other regions. **(B)** The transcriptional comparison of the SCN5A mutation/variation distribution between southern China and northern China, which was distinguished by before-DIII, after-DIII, and other parts of the SCN5A gene. The blue bar represents before-DIII parts. The orange bar represents after-DIII parts. The gray bar represents other parts.

Furthermore, the locations of SCN5A gene mutations/variants were divided into five parts as follows: N-Term, Transmembrane regions, IDL, C-Term, and others as shown in Figure 4A. The mutation/variation site distributions of SCN5A in southern China and northern China were 0, 50, 0, 30, and 20%, and 11.1, 24.5, 33.3, 8.9, and 22.2%, respectively (*p* = 0.000).

On the other hand, the locations of SCN5A gene mutations/variants were divided into three parts as follows: before-DIII, after-DIII, and others as shown in Figure 4B. The mutation/variation site distributions of SCN5A in southern China and northern China were 10, 80, and 10% and 60, 20, and 20%, respectively (*p* = 0.001).

**Figure 5:**
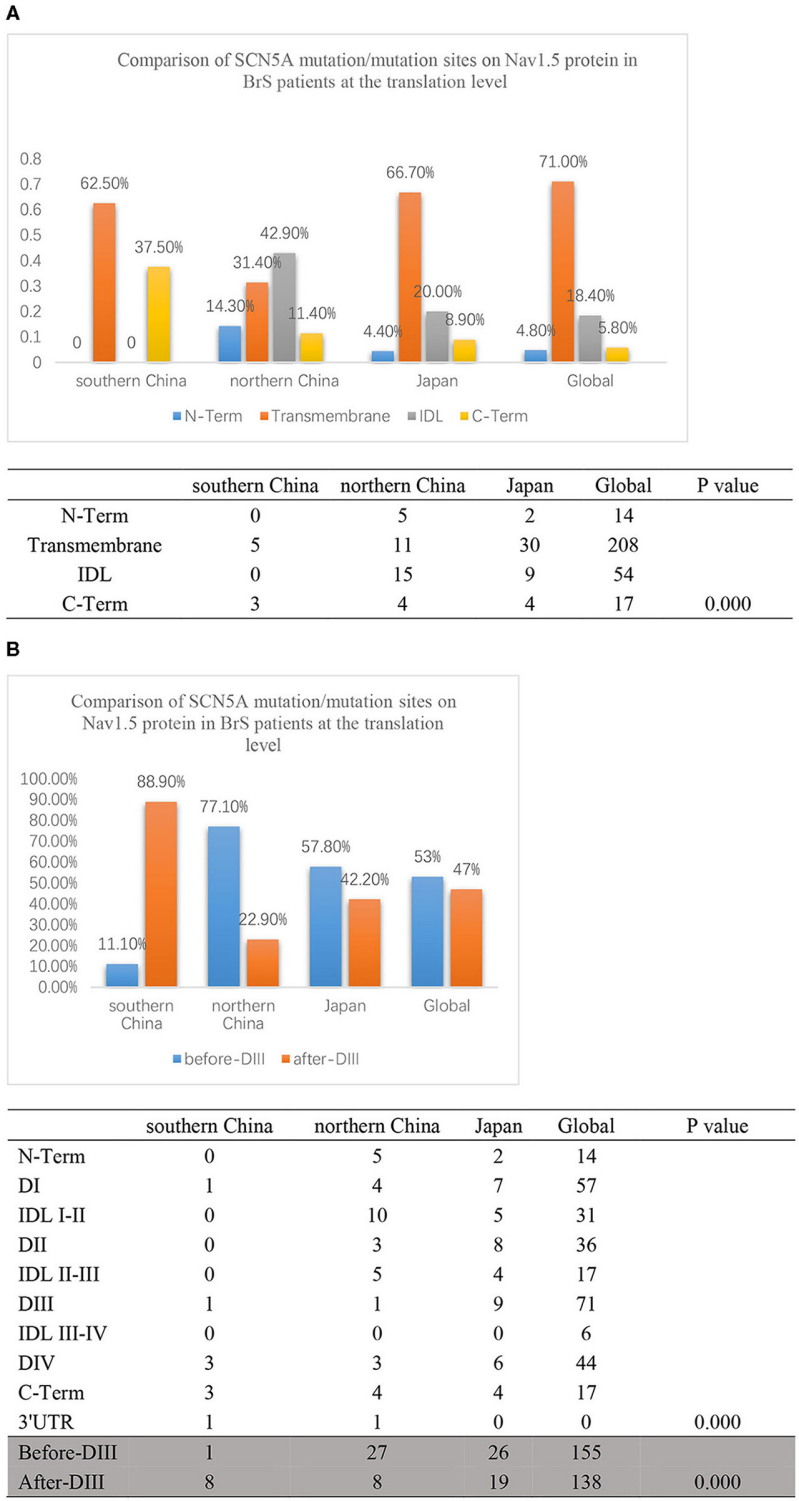
**(A)** The translation comparison of the SCN5A mutation/variation distribution on the Nav1.5 protein structure between southern China and northern China. Nav1.5 protein was distinguished by N-term, Transmembrane regions, IDL, C-term, and other parts. The light blue columns represent the N-terminus region. The orange columns represent the transmembrane region. The gray columns represent the IDL region. The yellow columns represent the C-terminus region. **(B)** The translation comparison of the SCN5A mutation/variation distribution on the Nav1.5 protein structure between southern China and northern China. Nav1.5 protein was distinguished by before-DIII and after-DIII. The blue columns represent before-DIII parts. The orange columns represent after-DIII parts.

Then, we compared the distributions of mutations/variations among China (southern China and northern China), Japan, and the world, as shown in Figure 5A. Japanese data refer to a Japanese multicenter register (42), and the global data are from the website http://triad.fsm.it/cardmoc/. We distinguished the SCN5A mutation sites on the protein Nav1.5 structure by N-term, Transmembrane regions, IDL, and C-term and found 0, 62.5, 0, and 37.5% for each part in southern China; 14.3, 31.4, 42.9, and 11.4% in northern China; 4.4, 66.7, 20, and 8.9% in Japan; and 4.8, 71, 18.4, and 5.8% in the world, respectively (*p* = 0.000).

In addition, the structure of Nav1.5 protein was divided into two parts: DI, IDL I-II, DII, and IDL II-III were set as the first half part (before-DIII), and DIII, IDL III-IV, DIV, and C-Term were set as the second half part (post-DIII) as shown in Figure 5B. The results indicated that 88.9% of the mutation sites were located in the post-DIII region in southern China, while only 22.9% in northern China, 42.2% in Japan, and 47% in the world (*p* = 0.000).

### PTMs Prediction for Nav1.5 Protein Change

As mutations that cause changes in amino acids may have influences on protein modification, we predictively analyzed the PTMs of SCN5A with software and mapped on the mutations/variations in China (Table 2), and found a tendency for amino acids to acquire more modification sites after mutation. PTM change was likely to occur in 72.7% of BrS patients from southern China and 26.7% from northern China (*p* = 0.000).

**Table 2:** The PTMs of SCN5A variants in Chinese BrS patients.

**Nucleotide change**	**Mutations**	**The potential PTMs before mutation**	**The potential PTMs after mutation**	**Structural position**	**Location**
-	A29A	-	-	N terminus	China
G87A	None	None	None	N terminus	China
-	Q55X	None	None	N terminus	China
G283A	V95I	None	None	N terminus	China
G292A	G98R	None	Methylarginine	N terminus	Gansu
-	A226V	None	None	DIS4	China
-	R230Q	None	Pyrrolidone carboxylic acid	DIS4	Xinjiang or Shanxi
T909-	M303T	None	O-linked_glycosylation, Phosphoserine	DIS5-S6	Xinjiang or Shanxi
-	K317N	None	N-linked_glycosylation	DIS5-S6	China
c.1198G>A	p.G400R	None	Methylarginine	DI S6	South China
-	V469V	-	-	DI-DII	Xinjiang or Shanxi
-	R511K	None	Ubiquitination	DI-DII	Xinjiang or Shanxi
-	V522A	None	None	DI-DII	Xinjiang or Shanxi
-	R535X	None	None	DI-DII	China
1651G>A	A551T	None	O-linked_glycosylation, Phosphoserine	DI-DII	China
A1673G	H588R	None	Methylarginine	DI-DII	China
1776C>G	N592K	N-linked_ glycosylation	Ubiquitination	DI-DII	China
c.1960G>T	p.E654X	None	None	DI-DII	China
-	H681P	None	Hydroxyproline	DI-DII	China
-	K698N	None	N-linked_glycosylation	DI-DII	Xinjiang or Shanxi
-	W822X	None	None	DII S4	China
-	G867X	None	None	DII S5-S6	China
-	G878G	-	-	DII S5-S6	Xinjiang or Shanxi
-	E1061E	None	None	DII-DIII	China
3269C>T	P1090l	None	None	DII-DIII	China
-	Q1118X	None	None	DII-DIII	China
C3549T	T1183T	-	-	DII-DIII	Hebei
G3578A	R1193Q	None	Pyrrolidone carboxylic acid	DII-DIII	China
4087insC	None	None	None	DIIIS4	South China
-	C1363F	None	None	DIII S5-S6	China
c.4282G>T	p.A1428S	None	Phosphoserine	DIII S5-S6	South China
-	delF1617	None	None	DIV S3-S4	China
-	R1623X	None	None	DIV S4	China
c.4886G>A	R1629Q	None	Pyrrolidone carboxylic acid	DIV S4	Fujian
C4946T	A1649V	None	None	DIV S4-S5	China
c.5262G>A	D1690N	None	N-linked_glycosylation	DIV S5-S6	Fujian
-	G1712C	None	S-palmitoyl_cysteine	DIV S5-S6	Guangxi
A5471G	N1774S	N-linked_ glycosylation	Phosphoserine	C terminus	Guangxi
-	S1812X	None	None	C terminus	China
-	D1818D	None	None	C terminus	China
c.5676delC	p.T1893P	None	Hydroxyproline	C terminus	South China
c.5692C>T	p.R1898C	None	S-palmitoyl_cysteine	C terminus	South China
-	R1913C	None	S-palmitoyl_cysteine	C terminus	China
-	V1951L	None	None	C terminus	China
6365a>G	None	None	None	3′UTR	China
C6995T	None	None	None	3′UTR	Fujian
7204t>A	None	None	None	3′UTR	China
7205c>T	None	None	None	3′UTR	China
703+130G>A	None	None	None	Intron 6	China
1143-3C>A	None	None	None	Intron 9	China
3840+73G>A	None	None	None	Intron 21	China
4245+81G>T	None	None	None	Intron 23	China
4245+82A>G	None	None	None	Intron 23	China
4299+83T>C	None	None	None	Intron 24	China
rs11708996 G>C	None	None	None	Intron	Taiwan, China

*Data from southern China are marked with a gray background*.

## Discussion

Our main findings in the study of the genetic characteristics of SCN5A in Chinese BrS patients were as follows: (1) More SCN5A gene mutations/variations were found in northern China than in southern China. (2) SCN5A mutations/variations of BrS patients in southern China mostly occurred in the DIII–DIV to C-terminus region and the 3′-UTR region of the SCN5A gene. (3) PTM changes were consistent with the mutation/variation distribution of SCN5A, which might be involved in the regulation of the pathogenesis of BrS patients.

BrS can be found all over the world, and the prevalence of BrS can reach 0.5%0 in high-prevalence areas. BrS is the leading cause of death for men less than 40 years old, only second to the death rate of traffic accidents in Southeast Asian countries (42, 43). In southern China, BrS patients were anticipated to have a relatively high incidence rate. However, our study revealed that SCN5A gene mutations published were found to be more in northern China than southern China. The possible reasons may be that BrS is a rare disease, and the total number of cases reported at present was not large and patients in many studies did not undergo DNA sequencing, which results in data bias. We will continue to pay attention to relevant reports and continue to collect cases to further confirm the data.

Further analysis showed that the locations of mutation sites had their own characteristics in southern China. Most mutation sites were clustered in the transmembrane regions in southern China statistically different from northern China. Mutation sites were mostly located in the second half part of the protein structure (post-DIII) in southern China, while in the first half part positions (before-DIII) in northern China, Japan, and the world.

The SCN5A gene, located on chromosome 3p21, contains 28 exons with a total length of about 80 kb and encodes the α-subunit protein Nav1.5. Some mutations lead to a decrease in current density, others do not lead to a decrease in I_Na_, while some location-specific SCN5A mutations resulted in poorer outcomes during follow-up (44). As different mutation locations lead to different pathological changes, we try to analyze whether protein functional modifications are involved in the mechanism.

PTM is a crucial modification method for protein transcription, such as phosphorylation, acetylation, ubiquitination, and glycosylation, which may bring a broad range of effects, such as protein stability, enzymatic activity, subcellular localization, and interactions. Multiple kinases including cyclic AMP-dependent protein kinase (PKA), protein kinase C (PKC), and calcium/calmodulin-dependent kinase II (CaMK II) phosphorylate regulate Nav1.5 channel physiology and pathology (45–48) including SUMOylation (49), ubiquitination (50), acetylation (51), etc. In our previous study, we revealed that miR-192-5p bound to the 3′-UTR of human SCN5A to negatively regulate the expression of Nav1.5 and reduce I_Na_ density. Our study demonstrated an important post-transcriptional role of miR-192-5p in post-transcriptional regulation of Nav1.5 (31).

Hence, we predicted the PTM sites with software and mapped on the mutations/variations in China. PTM change was likely to occur in 72.7% of BrS patients in southern China and 26.7% in northern China, suggesting that PTMs might be involved in the regulation of the pathogenesis of BrS, which provided new ideas and directions to further study the role of Nav1.5 in the pathogenesis of BrS.

## Conclusion

The mutation sites of BrS patients from southern China mostly distributed in the DIII–DIV to C-terminus region and the 3′-UTR region of the SCN5A gene, which was different from northern China, Asia, and other countries around the world. PTM change might be involved in the regulation of the pathogenesis of BrS.

## Contribution to the Field Statement

While BrS is a rare disease, it is especially young-male predominant and accounts for 20% sudden death without organic heart disease in Southeast Asia, which causes more and more concerns. We analyzed the genetic characteristics of SCN5A mutations/variations and found that SCN5A mutations/variations of BrS patients from southern China mostly occurred in the DIII–DIV to C-terminus region and the 3′-UTR region of the SCN5A gene, which was different from northern China, Japan, and the world. PTM changes predicted by the mutations/variations may be involved in the regulation of the pathogenesis of BrS. Our findings provide new ideas and directions to further study the role of Nav1.5 in the pathogenesis of BrS.

## Data Availability Statement

The original contributions presented in the study are included in the article/supplementary material, further inquiries can be directed to the corresponding author/s.

## Author Contributions

All authors participated in the design of the study, analysis and interpretation of the data, and review of the manuscript and approved the submitted version.

## Conflict of Interest

The authors declare that the research was conducted in the absence of any commercial or financial relationships that could be construed as a potential conflict of interest.

## Publisher's Note

All claims expressed in this article are solely those of the authors and do not necessarily represent those of their affiliated organizations, or those of the publisher, the editors and the reviewers. Any product that may be evaluated in this article, or claim that may be made by its manufacturer, is not guaranteed or endorsed by the publisher.
